# Every Prominent Man Seized with Pneumonia Dies

**Published:** 1906-05

**Authors:** 


EVERY PROMINENT MAN SEIZED WITH PNEUMONIA DIES.—So observes Abbott in a terse and pointed brief on the Treatment of Pneumonia, in his March journal. The rest is so frank and so full of truth that we quote a part verbatim: We mentally prepared Field's obituary the moment we heard he had a "slight cold." Wheeler's death was even surer. Given, a prominent man with pneumonia, a group of "eminent physicians" in attendance, each afraid to suggest any active therapeutic measure as the rest are sure to land on him with both feet, with a daily bulletin as the last straw, and the result is inevitable. Conscious that the eyes of the entire community are on them, they have more than one-half their attention fixed on the "grand stand," and the opportunities for effective intervention are unrecognized or unimproved; while the multiplicity of counsel renders the following of a consistent plan of treatment impossible.—*So. Med and Surgery.*

